# Eliciting preferences of patients about the quality of hospital services in the west of Iran using discrete choice experiment analysis

**DOI:** 10.1186/s12962-021-00319-y

**Published:** 2021-10-09

**Authors:** Ali Kazemi-Karyani, Vajiheh Ramezani-Doroh, Farid Khosravi, Zhila Seyedi Miankali, Shahin Soltani, Moslem Soofi, Maryam Khoramrooz, Behzad Karami Matin

**Affiliations:** 1 Research Center for Environmental Determinants of Health, Health Institute, Kermanshah University of Medical Sciences, Kermanshah, Iran; 2 Department of Health Management and Economics, School of Public Health, Hamadan University of Medical Sciences, Hamadan, Iran; 3 Student Research Committee, Kermanshah University of Medical Sciences, Kermanshah, Iran; 4 Social Development and Health Promotion Research Center, Health Institute, Kermanshah University of Medical Sciences, Kermanshah, Iran; 5 Center for Health Related Social and Behavioral Sciences Research, Shahroud University of Medical Sciences, Shahroud, Iran

**Keywords:** Hospital, Quality, Patient preferences, Discrete choice experiment, Iran

## Abstract

**Objectives:**

Knowing about accurate customer expectations is the most important step in defining and delivering high-quality services. This study aimed to evaluate the preferences of patients referring to two hospitals in Kermanshah, Iran.

**Method:**

Discrete choice experiment (DCE) method used to elicit preferences of 328 patients who were admitted in two hospitals of Kermanshah city in the west of Iran. Literature review and experts opinion were used to identify a candidate list of attributes related to the quality of cares in hospitals. The final study attributes were quality of physician care, quality of nursing care, waiting time for admission, cleaning of wards and toilets, and behavior of staff. Experimental design applied to extract choice sets of hospitals. The data was analyzed by a conditional logit regression.

**Results:**

The regression results showed the most important predictors of hospital selection by respondents was the good quality of physician care (aOR: 3.18, 95% CI 2.61, 3.87), followed by friendly behavior of staffs (aOR: 2.03, 95% CI 1.81, 2.27), cleanness of wards and toilet (aOR: 1.61, 95% CI 1.40, 1.85), and finally quality of nursing cares (aOR: 1.13, 95% CI 0.89, 1.44). However, increasing waiting time made disutility in the study participants (aOR: 0.69, 95% CI 0.60, 0.80).

**Conclusions:**

Our study finding emphasized some potential opportunity of quality augmentation in hospital sector by paying attention to different quality attributes including quality of physician, friendly behavior of staffs, cleanness of hospital environment and finally quality of nursing cares. Considering patients preferences in decision making process could lead to substantial satisfaction improvement.

## Introduction

Nowadays, quality is one of the important issues for managers of organizations, and this issue is especially considered in providing services. The quality of services is very effective in sustaining the performance of organizations and their survival, and the success of organizations depends on the optimal quality of the services [1, 2].

Therefore, organizations must strive to meet the demands of their customers. This will only be possible by moving towards customer orientation services. In fact, successful organizations are organizations that produce their products and services in a way that is in line with the wishes and preferences of costumers and have the necessary planning in place. On the other hand, customer satisfaction is a source of profit for organizations. Therefore, it is important to consider quality standards in service delivery. Also, the quality of goods and services has an impact on customer satisfaction and loyalty [3]. Modern management approaches have also defined quality as customer’s wishes and preferences [4].

In case of health care organizations, one of the most important missions of hospitals is to provide quality care to the referring patients and to meet their wishes and expectations. Meeting this requires the institutionalization of quality in hospitals [5]. In this regard, the National Health Service in the United States should consider a law that make it necessary to include customers’ preferences in their planning to provide services and reflecting client preferences in services provided is important item in evaluation plans of staffs [6].

Hospitals provide a set of similar services to their clients but quality of their services is different [7]. Thus, one of the differentiated aspects of hospitals is the quality of services and higher quality of services can be a suitable strategy to create competitive advantage in the hospital services market [8, 9].

Customer preferences are one of the most important determinants of customer evaluation of service quality and knowing about accurate customer expectations is the most important step in defining and delivering high-quality services. In fact, one of the current challenges of health systems is how to respond to patient expectations. Despite the importance of this issue, the recognition of patients’ preferences in providing care has often been neglected [10].

Iran heath system has a mixed structure of various providers (government, private, NGOs, charities) especially at the hospital level which majority of hospitals are allied to Ministry of Health and Medical Education [11]. There are different interventions aimed to increase hospital performance including accreditation of hospitals [12, 13]. Public hospitals are currently funded through a mixed system. The system includes government budgets, specific revenues and out-of-pocket payments by patients. The private hospitals are also financed through out-of-pocket payments and insurance reimbursements [14]. In this situation, attracting customers in public hospitals for reasons such as follows are important: (1) the implementation of an accreditation program that conducts an annual evaluation of the performance of hospitals that the ranking of hospitals and the budget allocated to them depends on it [15]; (2) a part of the income of hospitals is provided through special revenues and out-of-pocket payments which require people to go to public hospitals [14]; (3) the importance of equity in access to healthcare services with appropriate quality for all customers whether in the private or public sectors [16].

Due to the importance of quality of hospital services, some studies have been conducted in Iran to evaluate the quality of hospital services. For example, a study by Sabahi and colleagues examined the quality of hospital services from the perspective of patients in Kashan, Iran [17]. Another study examined the quality of services provided by private hospitals in Tehran [18]. These studies and other similar studies have been performed using the SERVQUAL model [19–21]. However, in recent years, some studies, have investigated the preferences of patients referring to hospitals and other health care centers in Iran and other countries using modern scientific methods such as conjoint analysis [22–25]. The advantage of this method is that it conforms to economic theories and the relative weight of attributes that affecting the expected quality of individuals can be extracted [26].

Regarding the importance of having information about the preferences of patients referring to hospitals and updating this information to improve the quality of hospital services, this study aimed to evaluate the preferences of patients referring to hospitals in Kermanshah, Iran. The findings of this study will help to identify preferences of patients for main attributes of quality of hospital services and to design the services according to patients’ preferences.

## Methods

This study used discrete choice experiment (DCE) method of analysis to elicit preferences of patients who were admitted in two hospitals of Kermanshah city in the west of Iran. DCE is a common method in market research for eliciting preferences of consumers in different areas of research. The basic assumption of this method is that we can describe a good or service with explain about its attributes [27]. In the recent decades, this method frequently has been used in health care to evaluate the preference of consumers and professionals of health systems [28]. In this study, three stages of DCE method were undertaken as follow [26]:

1. Identifying the attributes and attribute-levels: literature review [22, 23, 29, 30] and experts opinion were used to identify a list of attributes related to the quality of cares in hospitals. The primarily candidate list of attributes decreased to manageable numbers by expert opinion, too. levels of attributes selected according to literature review and opinion of research team. Table 1 shows attributes and attributes level included in final design of choice sets.
2. Experimental design: in this stage, scenarios made by experimental design based on D-efficiency method. Each choice set had two alternative scenarios that participants requested to select only one of them. To decrease cognitive burden for the patients, choice sets divided into 3 blocks with 7 choice sets. Also, for warm up and testing compatibility of responses, one dominant choice set were included in each block. After choice sets were designed, they included in a questionnaire that had three parts. The first part of the questionnaire involved demographic variables, education level, and information on hospital admissions, such as hospital type etc. the second part of questionnaire included questions to rating attributes of service quality from one (the lowest importance) to 10 (the highest level of importance). the third part of the questionnaire compromised definition of attributes and attribute- levels and one block of choice sets. there was three versions of the questionnaires that were different only in third part and participants randomly requested to one of them. then, the data of the questionnaired were pooled and analysed together.
3. Data analysis: the random utility model is the base of analysis DCE data. According to this theory, when person n should choose among j option, he/she choose the option that produces more utility. in this study, the utility of a hospital had two elements. The first part was the random element of utility (Vni) that is derived from attributes of hospital services ( x1,…., x5) that are visible and measurable. The second element is unexplained part of utility (ɛ_ni_). the conditional logistic model performed to estimate the utility of attributes of service quality in hospitals follows:

$$U_{{ni}} =V_{{ni}} + \varepsilon_{{ni}} = \alpha_{1} + \beta_{1} x_{{1ni}} + \beta _{2} x_{{2ni}} +\cdots + \beta _{m} x_{{mni}} +{\varepsilon_{ni}}$$

where $U_{{ni}}$ is the utility of scenario i for person n, coefficients (β_1,_…β_m)_ are the utility of attributes-levels and prediction of accepting one scenario.

In order to analyze the data, in addition to the analysis using conditional logit model for the whole sample, separate conditional logit models for different subgroups of sex (female, male), education level (diploma and lower, academic), residence place (urban, rural) and hospital (Imam Reza, Shohada) were also performed. Also, in addition to the DCE study we asked participants of the study to rate final attributes in a scale of 1 to 10. 

### Study population and sampling

The study population was patients hospitalized in hospitals of Kermanshah city, western Iran. Two hospitals included in the study, a public hospital (Imam Reza) affiliated to Kermanshah University of Medical Sciences, and a hospital affiliated to Social Security Insurance organization (Shohada), that directly provides hospital services for its insurers. Imam Reza and Shohada hospital had about 750 and 110 active beds, respectively in 2019. From both hospital three-hundered-twenty-eight patients were included in the study. Simple random method of sampling was used to select patients based on number of beds. Inclusion criteria for the patients were above 15 years old, consent for participation in the study and cognitive ability to respond to the questionnaire. Data were gathered with face to face interview with patients.

**Table 1:** Attributes and attributes-levels included in the final design of choice sets

Attribute	Level (definition)
Waiting time for admission	1 hour, 2 hours, 3 hours and more
Quality of physician care	Low (physician has not a friendly approach with patient. Unlikely he/she provides the patient with the necessary information about the disease, diagnostic tests and treatment), moderate (physician has a friendly approach with patient. He/she provides the patient with the necessary information about the disease, diagnostic tests and treatment. He/she might have any other good qualities), good (physician has a friendly approach with patient. He/she provides the patient with the necessary information about the disease, diagnostic tests and treatment. He/she also involves the patient in making decisions) [31]
Quality of nursing care	Low (nurses have not a friendly approach with patient. Unlikely, they provide patient with understandable information about the patient care), moderate (nurses have a friendly approach with patient. They provide patient with understandable information about the patient care), good (nurses have a friendly approach with patient. They provide patient with understandable information about the patient care and reassurance for the patient. they also involves the patient in making decisions) [31]
Cleaning of wards and toilets	Often clean, almost clean, always clean
Behavior of staff	Indifferent, friendly

## Results

Two hundred and forty-eight (75.61%) patients from Imam Reza hospital and 80 patients from Shohada hospital were included in the study. Of a total of 328 participants, 148 (45.12%) were women. The mean age of the patients was 45.15 (standard deviation [SD] = 17.68) years and about 73% had a high school diploma or lower. Most of patients (74.9%) were from urban areas.

**Fig. 1:**
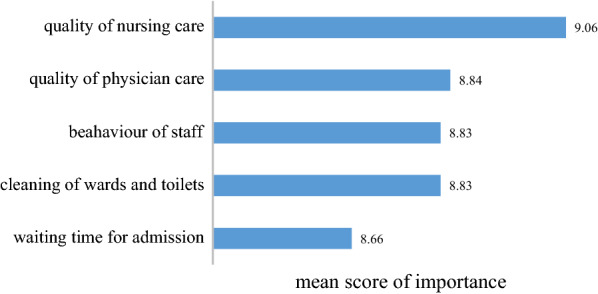
Opinion of patients about importance of hospital services quality attributes

The survey of patients opinion about the importance of hospital service quality attributes showed that the quality of nursing services scored an average of 9.06 out of 10. Quality of medical care came in second importance rank with a mean score of 8.84. The least important rank was belonged to the waiting time at admission, which averaged 8.66. Figure 1 shows mean score of importance of included attributes from perspective of patients in a scale of 1 to 10.

### Findings from discrete choice experiment model

The findings of DCE model showed that the physician care was the most desirable attributes, so improving the quality of these services from a low level to a good level increased the odds ratio of hospital selection by 3.18 times (95% CI 2.61, 3.87). Increasing waiting time for patients would be disadvantageous and increasing the waiting time reduced the chance of hospital selection for receiving a treatment. The odds ratio of good level of nursing services’ quality was 1.13 which was not statistically significant (p < 0.1). Improving the cleaning of wards and toilets would create utility and increase the odd ratio of hospital choice. Enhancement of hospital cleanliness from the “often clean” to “almost clean” increased the probability of hospital being selected by 61%. Also, in comparison with indifferent behavior of staff, the friendly behavior make utility for participants and significantly increased the odds ratio of hospital selection (aOR: 2.03, 95% CI 1.81, 2.27). Also, the fitted model was statistically significant (P < 0.001). Table 2 shows patients’ preferences about the quality of hospital services in Kermanshah province, Iran in 2019.

**Table 2:** Preferences of participants of the study about the attributes of service quality in Kermanshah, Iran, 2019

Choice	aOR	95% CI	p-value
Waiting time for admission (base: 1 h)			
2 h	0.69	(0.60, 0.80)	< 0.001
3 h and more	0.90	(0.76, 1.05)	0.187
Quality of physician care (base: low)			
Moderate	1.31	(1.13, 1.50)	< 0.001
Good	3.18	(2.61, 3.87)	< 0.001
Quality of nursing care (base: low)			
Moderate	0.77	(0.61, 0.96)	< 0.050
Good	1.13	(0.89, 1.44)	0.305
Cleaning of wards and toilets (often clean)			
Almost clean	1.61	(1.40, 1.85)	< 0.001
Always clean	1.05	(0.88, 1.25)	0.591
Behavior of staff (base: indifferent)			
Friendly	2.03	(1.81, 2.27)	< 0.001
Observations	5904.00		
Prob > chi^2^	< 0.001		
Pseudo R^2^	0.16		

aOR: adjusted odds ratio; 95% CI: 95% confidence interval

### Subgroup analysis

Women attained more utility from improving the level of physician care than men and increasing quality of physician cares from “low” to “good” level, increased odds ratio of choosing hospital about 4 times that this figure is 2.78 for men. However, utility of nursing care in men (aOR: 1.48, 95% CI 1.06, 2.07) was higher than those in women (aOR: 0.81, 95% CI 0.57, 1.15). Also, the cleaning of wards and toilets made higher utility for men. In case of behavior of staff, this attribute had higher importance and utility for women. Disutility of waiting time was higher in participants with academic education (aOR: 0.64, 95% CI 0.47, 0.87). In all education level, physician care with good quality made higher utility than other attributes of hospital care.

People with low education received more utility from nursing services than those with higher education. The waiting time for admission for hospitalized patients at Shohada Hospital had more disutility than Imam Reza hospital patients. The utility of good physician services for hospitalized patients at Shohada hospital was much higher than for hospitalized patients at Imam Reza hospital (aOR: 4.67 vs. aOR: 2.87). Table 3 shows the findings for subgroup analysis.

**Table 3:** Preferences of different subgroups of participants about the attributes of service quality in Kermanshah, Iran, 2019

Choice	Gender		Residency		Education level		Hospital	
	Female	Male	Urban	Rural	Diploma and lower	Academic	Imam Reza	Shohada
	aOR (95% CI)	aOR (95% CI)	aOR (95% CI)	aOR (95% CI)	aOR (95% CI)	aOR (95% CI)	aOR (95% CI)	aOR (95% CI)
Waiting time for admission (base: 1 h)								
2 h	0.58 (0.46, 0.73)	0.76 (0.63, 0.92)	0.66 (0.55, 0.78)	0.77 (0.58, 1.02)	0.80 (0.68, 0.95)	0.44 (0.33, 0.59)	0.73 (0.62, 0.87)	0.54 (0.39, 0.74)
3 h and more	0.98 (0.77, 1.25)	0.82 (0.66, 1.02)	0.85 (0.70, 1.02)	1.04 (0.75, 1.44)	1.01 (0.84, 1.22)	0.64 (0.47, 0.88)	1.08 (0.89, 1.29)	0.48 (0.34, 0.68)
Quality of physician care (base: low)								
Moderate	1.16 (0.94, 1.44)	1.48 (1.22, 1.79)	1.26 (1.06, 1.48)	1.49 (1.12, 1.97)	1.34 (1.13, 1.58)	1.23 (0.93, 1.63)	1.30 (1.10, 1.53)	1.33 (0.98, 1.79)
Good	3.99 (2.91, 5.48)	2.78 (2.15, 3.60)	3.48 (2.75, 4.40)	2.48 (1.70, 3.60)	3.09 (2.46, 3.88)	3.64 (2.46, 5.38)	2.87 (2.30, 3.58)	4.67 (2.98, 7.32)
Quality of nursing care (base: low)								
Moderate	0.49 (0.35, 0.69)	1.08 (0.79, 1.48)	0.75 (0.58, 0.98)	0.80 (0.51, 1.26)	0.89 (0.69, 1.17)	0.47 (0.30, 0.74)	0.82 (0.63, 1.06)	0.60 (0.36, 0.97)
Good	0.81 (0.57, 1.15)	1.48 (1.06, 2.07)	1.12 (0.84, 1.47)	1.19 (0.74, 1.93)	1.34 (1.01, 1.7)	0.69 (0.43, 1.11)	1.22 (0.92, 1.56)	0.87 (0.51, 1.46)
Cleaning of wards and toilets (often clean)								
Almost clean	1.65 (1.32, 2.05)	1.57 (1.31, 1.89)	1.68 (1.43, 1.98)	1.46 (1.12, 1.91)	1.55 (1.31, 1.82)	1.86 (1.41, 2.44)	1.54 (1.32, 1.81)	1.88 (1.40, 2.54)
Always clean	0.83 (0.63, 1.10)	1.24 (0.98, 1.56)	1.01 (0.82, 1.23)	1.22 (0.87, 1.73)	1.01 (0.82, 1.22)	1.19 (0.84, 1.70)	0.98 (0.81, 1.20)	1.30 (0.88, 1.91)
Behavior of staff (base: indifferent)								
Friendly	2.26 (1.90, 2.69)	1.91 (1.65, 2.22)	2.00 (1.76, 2.29)	2.11 (2.62)	2.13 (1.87, 2.44)	1.78 (1.44, 2.22)	1.99 (1.76, 2.27)	2.16 (1.69, 2.75)
Observations	2664	3240	4374	1530	4320	1584	4464	1440
Prob > chi^2^	< 0.001	< 0.001	< 0.001	< 0.001	< 0.001	< 0.001	< 0.001	< 0.001
Pseudo R^2^	0.20	0.15	0.17	0.14	0.16	0.19	0.14	0.24

aOR: adjusted odds ratio; 95% CI: 95% confidence interval

## Discussion

Overall respondents of present study were willing to wait for more time in exchange for more quality of physician care, friendly behavior of staffs and more clean wards and toilets.

Regarding the findings of the rating of the attributes in a scale of 1 to 10, the studied attributes had an order as follow: nursing care quality, physician care quality, staffs behavior, clean wards and toilets, and finally waiting time.

The conditional logit analysis revealed that the most important attributes in the preferences of respondents was the quality of physician care. The result is in line with other studies findings [29, 31–35] In Iran, most of well-known physician work at public sector hospitals. It seems patients seek treatment in these hospitals to utilize from high quality care of these physicians. However, other studies found that waiting time was the most important attributes in the decision making of their population, the difference in the setting and the studied population could be the probable reason for this dissimilarity [22, 23, 36]. In other word, in the present study respondents were hospitalized and could wait for more time to receive services.

The second most crucial and statistically significant characteristic in the utility function of surveyed individuals was behavior of staffs. In line with other studies, patients preferred that hospital staffs treat them friendly [22, 37–39]. However, another study in Iran showed different results. Bahrampour et al. found that the attitude of staff was not a statistically significant factor in the utility function of their participants [34]. Maybe the differences between two populations and the study setting are the reasons of this discordance.

As previous expectation, cleanness of wards and toilet was another hospital quality attribute that would increase the odds ratio of hospital attendance by individuals that is aligning with the previous studies [22, 30, 34, 40]. O’Hara et al. [40], elicited preferences for hospital quality metrics of hip and knee arthoplasty patients. Their results revealed that the cluster of patients with more insurance coverage and less pain period preferred hospitals with lower complication rates compared to national standards [40]. The likely cause of our finding could be the relationship between hospital infection risk and cleanness of hospital. Another possible reason for it could be the length of stay in hospital. Hospitalized patients compared to outpatient ones stay for longer time in hospital and it seems to be more sensitive to the purity and disinfection of their environment.

Increasing waiting time would decrease the odds ratio of hospital selection. Our findings is parallel with the previous findings [22, 34, 38, 41]. Our studied individuals were willing to wait for more time to receive high quality care of physician that supports the result of Marshall et al. Their study showed that patients were willing to trade-off between waiting time and reputation of surgeon [38]. Another study in Iran showed parallel result that many of patients preferred to wait for more time and pay more cost to receive higher quality services [22]. However the most important attributes in individuals decision was not waiting time and it is like other studies findings [29, 31, 34] but as stated above in Iran repetitious physician work at governmental hospitals and it seems patients prefer to forgone their time in order to meet famous physician at these hospitals as in the Rubin et al. study, for some services patients were willing to wait for more time to visit a physician that they wanted [42].

Quality of nursing cares was the last influential attributes. This attributes surprisingly had a negative utility score and indicating by increasing the quality of services delivered by nurses from the low to the moderate level the odds ratio of selection hospitals by individuals would be decrease. Preferences with high variation in this attributes may be one probable reason for this finding.

Subgroup analysis revealed women compared to men gained more utility from increasing the quality of physician cares. While regarding the quality of nursing cares, men gained more utility compared to women. This is probably because of this that women use more preventive and diagnostic services while men use emergency services the most [43]. Increasing waiting time for both groups would lessen their utility. Improving cleanness of environment and friendly behavior of hospital staffs had a positive relationship with on hospital selection by two groups, too. However in the latter group the size of the relationship for women was larger, this could be because of more sensitivity of women regarding behavior of others. As in another study by Kurk et al. found that one of the leading factors for women in selection a baby delivery health facility was staff attitude [39].

Regardless of whether patients were high or low educated, they preferred receiving a high level of care quality by physicians. However regarding the quality of nursing care, less educated patients compared to more ones gained more utility from upsurge in these cares. One probable reason for this finding could be expectations. More educated individuals expected excellent level of quality and were not tolerant of the qualities levels.

From patient point of view there were not any outstanding differences between urban and rural patients regarding various quality attributes. Both groups preferred a hospital that had physician with better quality services, was cleaner, and had welcoming staffs. The probable reason could be equal access to information sources and as a result close expectations between rural and urban areas. However there was two dissimilarities in their utility functions. First, waiting time only caused disutility for urban groups and it did not has any statistically significant effects on rural respondents. The possible reason could be time limitation of urban subgroup. Second, the odds ratio of hospital attendance by urban populations decreased as the quality of nursing care increased.

Subgroup that was differentiated by Shohada hospital dis-utilized from extended waiting time from 1 to 2 hours and from 2 to 3 hours but respondent of Imam Reza hospital did not prefer hospitals that its waiting time increased from 1 to 2 hours. Regarding physician care quality, increasing the quality from low to moderate and from moderate to good created utility for Imam Reza hospital patients but in Shohada hospital only going from moderate level to good one could increase the odds ratio of selecting a hospital. It seems the expectations of patients of social security affiliated hospital were more than other hospital respondents. The quality of nursing care had a statistically significant effect on Shohada participants in a way that with increase of the quality level from low to moderate the odds ratio would decrease. The other two attributes, cleanness of wards and toilet and staff behavior had same effects on these two subgroups and increased the odds ratio of hospital selection.

Although the present study was the first attempt to elicit Kermanshah residents’ preferences for hospital quality aspects using DCE, but there were several limitations in it and the results should be interpreted with caution. First, there were other quality attributes that we did not include in our study. Hospital quality indicators are a wide range of attributes and at this study we only studied a small subset of them. Further research to include other indicators seems necessary. Second, other influential groups’ preferences like physician did not surveyed. Third, the participant in this study came from a city in Iran and we could not generalize its result to the whole of country.

## Conclusions

One of the most important steps forward to improve quality of hospital services is to elicit consumer preferences, ensure shared decision making and finally increase patient satisfaction. Our study finding emphasized some potential opportunity of quality augmentation in hospital sector by paying attention to different quality attributes including quality of physician, friendly behavior of staffs, cleanness of hospital environment and finally quality of nursing cares. Considering patients preferences in decision making process could lead to substantial satisfaction improvement.

## Data Availability

The corresponding author (BKM) and another investigator (AKK) have access to the dataset. The data set analyzed during the current study is available from the corresponding author on reasonable request.

## Abbreviations

SERVQUAL: Service quality
DCE: Discrete choice experiment
